# Determinants of Maternal Health-Related Quality of Life after Childbirth: The Generation R Study

**DOI:** 10.3390/ijerph16183231

**Published:** 2019-09-04

**Authors:** Guannan Bai, Ida J Korfage, Eva Mautner, Hein Raat

**Affiliations:** 1 Department of Public Health, Erasmus MC-University Medical Centre Rotterdam, Wytemaweg 80, Rotterdam, 3015 CN, South Holland, The Netherlands; 2 The Generation R Group, Erasmus MC-University Medical Centre Rotterdam, Wytemaweg 80, Rotterdam, 3015 CN, South Holland, The Netherlands; 3 Department of Obstetrics and Gynecology, Medical University of Graz, Auenbruggerpl. 2, Graz 8036, Austria

**Keywords:** childbirth, community sample, determinants, quality of life, women

## Abstract

Having good health-related quality of life (HRQoL) is essential, particularly for women after childbirth. However, little is known about its determinants. We aimed to identify the determinants of HRQoL after childbirth in a large community sample in the Netherlands. We have included 4312 women in the present study. HRQOL was assessed by a 12-Item Short Form Survey (SF-12) at around two months after childbirth; Physical and Mental Component Summary scores were calculated. Information on 27 potential determinants of HRQoL was collected through questionnaires and medical records. Multivariate linear regression models were applied to assess significant determinants of physical and mental HRQoL. Our study showed that older maternal age, shorter time since childbirth, elective/emergency cesarean delivery, loss of energy, maternal psychopathology, and the hospital admission of the infant were significantly associated with worse physical HRQoL (*p* < 0.05); older maternal age, non-western background, low household income, loss of energy, and maternal psychopathology were significantly associated with worse mental HRQoL (*p* < 0.05). We identified multiple determinants of suboptimal physical and mental HRQoL after childbirth. In particular, maternal psychopathology after childbirth was profoundly associated with mental HRQoL. These women may need support. We therefore call for awareness among health care professionals.

## 1. Introduction

Every minute about 255 infants are born worldwide [1]. The birth of a child may impact a woman’s physical, psychological, and social health. In past decades, the focus of maternity care in the developed countries has expanded from the traditional goal of reducing mortality and morbidity to broader aims, such as improving health-related quality of life (HRQoL) [2]. HRQoL is a multidimensional concept that incorporates physical, psychological, and social domains of health [3], which is in accordance with the definition of health by the World Health Organization as ‘not merely the absence of disease or infirmity but a state of complete physical, mental and social well-being’ [4].

Giving birth by a woman and adaptations during weeks after childbirth may have a significant impact on women’s HRQoL. Issues mentioned in the literature such as urinary incontinence and other common health problems may affect HRQoL [5,6,7,8,9,10,11,12,13]. In addition, women’s HRQoL after childbirth may be affected by factors related to pregnancy and delivery [12,14,15], by infant’s health [16], and by non-medical factors, such as race, maternal age, and socioeconomic status [10,17,18]. For example, Oliveira et al. found that mothers who were white, 30–40 years of age, and had relatively high education were more likely to report relatively good HRQoL [10]. Relatively high maternal age may increase the risk of some adverse physical and mental health outcomes [19,20], which may impair HRQoL. Insight in the factors associated with women’s HRQoL after childbirth is needed to develop appropriate health interventions [9]. However, associations between some factors, such as maternal age, mode of delivery, and HRQoL after childbirth are not consistent across studies [12]. Most studies only focused on limited numbers of potential determinants of HRQoL after childbirth, and most of these findings were based on relatively small sample sizes. A comprehensive overview of determinants is lacking.

In this study, we investigated a broad set of potential determinants of HRQoL after childbirth in a large community sample. As potential determinants, we included factors related to both pregnancy and delivery, to common health problems after childbirth, to infant’s health, and non-medical factors of mothers and infants. 

## 2. Materials and Methods 

### 2.1. Data Source

The present study was embedded in the Generation R study, a prospective population-based study spanning fetal life to adulthood. The Generation R study has been described in detail elsewhere [21,22,23]. Briefly, the cohort study includes 9778 (response rate 61%) mothers and their children born between April 2002 and January 2006 in the Rotterdam area, the Netherlands [23]. The data was collected through several sources: questionnaires that were completed by women during pregnancy and at two months after childbirth, hospital medical records in the hospital and midwives practices, and by fetal ultrasound examinations. Questionnaires were posted to the participants by study staff members. Fetal ultrasound examinations were performed at each prenatal visit. Protocols for data collection were followed. More details about data collection have been described elsewhere [21,22,24]. 

The study was conducted in accordance with the World Medical Association’s Helsinki guidelines [25], and was approved by the Medical Ethical Committee of the Erasmus MC-University Medical Center Rotterdam (approval number: 217.595/2002/203; the date of approval: 9 January 2003). Written consent had been obtained from all participating women. 

### 2.2. Study Population

**Figure 1:**
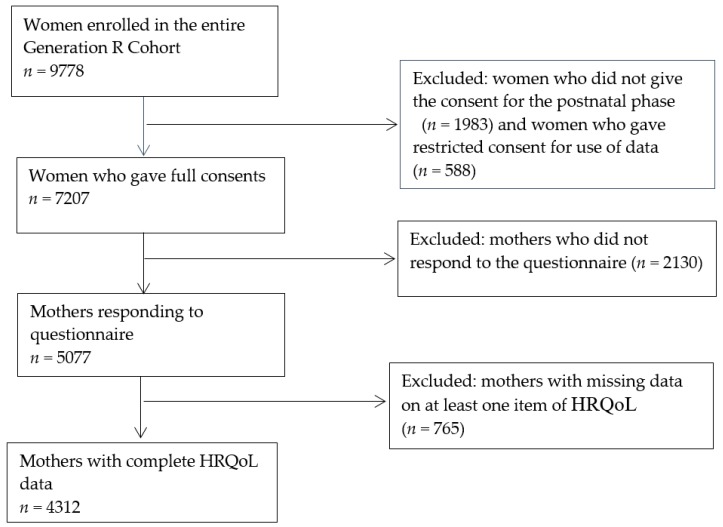
Flow chart of the study population.

As shown in Figure 1, we have included 4312 mothers who were eligible for analyses in the present study. 776 (18%) mothers had multiple pregnancies or births included in the generation R study. Information related to the first pregnancy and the first child per mother was selected for analyses in this study. 

### 2.3. HRQoL

Maternal HRQoL at two months after childbirth was assessed by the 12-Item Short Form Survey (SF-12); the recall period was one month [26]. There are eight scales: physical functioning, role limitations due to physical problems, bodily pain, general health, vitality, social functioning, role limitation due to emotional problems, and perceived mental health [26]. To calculate the Physical Component Summary (PCS) score and the Mental Component Summary (MCS) score, we followed the standard procedure as described in the SF-12 user manual [27]. A higher score indicates a better HRQoL.

### 2.4. Potential Determinants

Based on the literature cited in the Introduction, we selected the following variables as the potential determinants of maternal HRQoL after childbirth.

#### 2.4.1. Mother and Infant Demographic Characteristics

Maternal age at enrolment, ethnic background, educational level, marital status, and household income were collected by the questionnaire when women were enrolled in the Generation R study. Maternal ethnic background and educational level were defined according to the classifications of tatistics Netherlands [28,29]. Ethnic background was categorized into three categories: native Dutch, other western immigrants, and non-western immigrants [28]. Education was categorized into four successive levels based on the Dutch standard classification of education: high (Master’s degree or Ph.D.), mid-high (higher vocational training, Bachelor’s degree), mid-low (>3 years general secondary school, intermediate vocational training), and low (no education, primary school, lower vocational training, intermediate general school, or three years or less general secondary school) [29]. Marital status was coded as single and married/living together. Household income was coded as low (<2200 euros per month) and high (≥2200 euros per month). 

Time since childbirth (in months) was reported by mothers when they filled in the postnatal questionnaire at two month after childbirth. Infant’s gender was based on medical record from the hospital and midwives practices.

#### 2.4.2. Characteristics of this Pregnancy

Information on parity, unplanned pregnancy, hospitalization during pregnancy, and gestational weight gain [30] was obtained by the questionnaires. Information on the twin birth and pregnancy complications (i.e., pregnancy-induced hypertension, pre-eclampsia, and gestational diabetes) [31] was obtained from the medical records from hospital and midwives practices. 

#### 2.4.3. Delivery Characteristics

Mode of delivery was divided into four categories; (1) spontaneous vaginal delivery; (2) induced vaginal delivery (including expression, forceps, and vacuum extraction); (3) elective cesarean delivery; (4) emergency cesarean delivery. Location of delivery was categorized into three categories: (1) at home; (2) at hospital; and (3) at child birth clinic or other places. 

#### 2.4.4. Maternal Health-Related Factors after Childbirth

Maternal health-related factors after childbirth were loss of energy, headache, and maternal psychopathology (score in tertile) which were assessed by the postnatal questionnaire at two months. Maternal psychopathology was measured using the Global Severity Index (GSI) of the Brief Symptom Inventory (BSI) [32]. The total GSI score was divided into tertiles using 3 and 10 as cut-off points [33]. 

#### 2.4.5. Infant Health-Related Factors

Information on meconium-stained amniotic fluid (yes or no), Apgar score at 5 min after childbirth (below seven; eight or higher), birth weight (<2500 g, ≥2500 g), and intrauterine growth restriction was obtained from the medical records in the hospital and midwives practices. Gestational age was determined by the fetal ultrasound examination. Preterm birth was defined as the birth before 37 weeks of gestation. Small for gestational age was defined based on standard deviation curves derived from the Generation R birth cohort [24]. Hospital admission in the first week after birth was measured in the postnatal questionnaire at two month after childbirth. 

### 2.5. Statistical Analyses

Descriptive analyses were applied to characterize the population for analyses (*n* = 4312). The differences in the mean Physical and Mental Component Summary scores across categories of potential determinants were assessed by two independent sample t-tests and one-way analysis of variance (ANOVA). The variables that were found to be significant in the above step were then included in the final regression models, as well as maternal age at enrollment and time since childbirth. Multivariate linear regression was applied to assess the significant determinants of physical and mental HRQoL after childbirth. Multiple imputations were applied to deal with the missing data. Imputations were based on the relationships between all variables [34]. Five imputed datasets were generated. Additionally, we also conducted multivariate linear regression analysis using the non-imputed data. The clinical relevance was assessed by effect size (Cohen’s d). Cohen’s d was calculated by dividing the difference in mean scores between subgroups by the largest standard deviation and interpreted as: 0.2 ≤ d < 0.5 small difference, 0.5 ≤ d < 0.8 moderate difference, and d ≥ 0.8 large difference [35].

We also assessed the differences between the study population (*n* = 4312) and the population excluded from analyses.

All analyses were conducted in SPSS 21.0 (IBM Corp., Armonk, NY, USA). Significance was indicated at *p* < 0.05.

## 3. Results

### 3.1. General Characterisitics of Mothers and Children

4312 mothers were included in the study. Table 1 shows the characteristics of mothers and infants. The mean maternal age at enrollment was 31 years. Half of the women had a bachelor’s degree or above educational level (i.e., high and mid-high education). More than 60% of mothers were Dutch. For about 60% of mothers it was their first child; almost 80% had a spontaneous vaginal delivery; and 80% delivered at hospital. 5% of mothers gave a preterm birth. 

### 3.2. Multivariate Regression Analysis

We applied the bivariate analyses to select the statistically significant categorical variables (see Supplementary Table S1) to be included in the multivariate regression models. Table 2 and Table 3 shows the coefficient betas and corresponding 95% confidence intervals and *p* values in the multivariate regression analyses based on imputed data. Older maternal age at enrolment, shorter time since childbirth, low household income, elective/emergency cesarean delivery, loss of energy, maternal psychopathology, and the hospital admission of the baby were significantly associated with lower physical component summary scores (*p* < 0.05). Older maternal age at enrolment, non-western background, low household income, unplanned pregnancy, loss of energy, headache, and maternal psychopathology were significantly associated with lower mental component summary scores (*p* < 0.05). In addition, Supplementary Table S2 presents the coefficient betas and corresponding 95% confidence intervals and *p* values based on the non-imputed data. The patterns of significant determinants are similar in both datasets.

### 3.3. Non-Response Analyses

Supplementary Table S3 shows the differences between the study population (*n* = 4312) and the population excluded from analyses (*n* = 5466). Compared with the excluded women, the study population more often had higher social economic status and better health.

## 4. Discussion

We have assessed an extensive set of potential determinants of maternal HRQoL at two months after childbirth in a large, population-based sample of women in the Netherlands. Multiple factors were found to be significantly associated with worse physical and mental HRQoL after childbirth. 

In interpreting our results, it is important to be aware that a statistically significant difference does not necessarily indicate clinical relevance. In the present study, we indicated the clinical relevance of findings using Cohen’s effect size (d); the difference in the mean physical and mental HRQoL scores is clinically relevant when d ≥ 0.5. Based on this classification, the clinical relevance of most of the significant differences in our study can be considered small. There are a few exceptions, which we will describe in somewhat more detail. The difference in the mean score of physical HRQoL after childbirth between women having elective cesarean delivery and those having spontaneous vaginal delivery can be interpreted as clinically relevant (d = 0.49). Cesarean delivery is a surgery operation that can cause pain, discomfort, and other health symptoms. Therefore, women may perceive their physical health as worse than that of women who had a vaginal delivery [36,37]. Martínez-Galiano et al. found that cesarean delivery was associated with a worse overall HRQoL score at six weeks after childbirth in a large sample of Spanish women [8]. The potential effects of a cesarean delivery on HRQoL after childbirth may be taken into consideration when deciding about cesarean delivery. 

A notable clinically relevant finding in our study is the large difference of 14 points between the mean score of mental HRQoL between mothers with most psychopathological symptoms (i.e., defined as highest tertile) and those with least psychopathological symptoms (i.e., defined as lowest tertile). Though adjusted by other variables, results from the regression analyses show that the difference between the above two groups is still large in terms of clinical relevance; maternal psychopathology after childbirth was profoundly associated with worse mental HRQoL. Our finding is consistent with previous studies [9,38]. Emmanuel et al. found that even when they controlled for other confounding variables, having psychopathological symptoms was significantly associated with lower HRQoL in the period from late pregnancy to 12 weeks after childbirth [38]. Martínez-Galiano et al. found that a significant decrease in HRQoL occur in women with depressive symptoms and anxiety [9]. In the early postpartum period, psychopathological symptoms have been shown to affect women’s ability to function, mental health status, inter-personal relationships, social engagement, and overall quality of life [39]. Women with postpartum psychopathological symptoms may need support. We therefore call for awareness among health care professionals. 

In the present study, longer time since childbirth was associated with better physical HRQoL, indicating women’s physical HRQoL improved over time. This finding is consistent with the natural course of recovery after childbirth.

Factors associated with maternal HRQoL after childbirth reported by previous studies, such as multiparity, more gestational weight gain [6], preeclampsia [40], and gestational hypertension [15], were not confirmed in the present study. The reason why our results are not consistent with a number of previous studies may be related to the use of a variety of instruments to measure HRQoL. In addition, the health conditions (e.g., gestational hypertension and pre-eclampsia) in our study may be less severe than those in other studies, because women participating in the Generation R study are, in general, more healthy in comparison with women participating in the clinical studies.

In the present study, we aimed to assess HRQoL at two months after childbirth. However, the questionnaire was completed between 2 weeks and 6 months postpartum. Twenty-five percent of women filled in the postnatal questionnaire within two months after childbirth; 50% during 2–3.5 months; and 25% after 3.5 months after childbirth. In case of a longer interval after childbirth, HRQoL may have reached the pre-pregnancy level. To check this issue, we compared the mean Physical and Mental Component Summary scores with the Dutch normative data of women aged 30–39 years old. We found that the mean Physical Component Summary score in our study was below the normative data (*p* < 0.001), indicating that women still had not fully recovered physically [41]. The mean Mental Component Summary score in our study was similar to the normative data (*p* = 0.28). 

The major strengths of this study are the large sample size and the availability of an extensive set of potential determinants of maternal HRQoL after childbirth including socio-demographic characteristics, pregnancy- and delivery-related factors, maternal health-related factors after childbirth, and infant’s health-related factors.

There are several limitations that need attention.

First, even though we aimed to include as many potential determinant variables as possible, still some relevant factors were not measured. This applies, for instance, to symptoms that may occur after childbirth such as difficulties in breastfeeding, urinary incontinence, fecal incontinence, constipation, sleeping difficulties, and back pain [5,6,38,42]. Also, women’s difficulties in sexual intercourse may be important causes of unhappiness for women [9,42]. Problems with the couple’s relationship dynamics and the social support can also affect overall HRQoL and mental HRQoL [9,43]. These factors were not included in our analyses since these factors have not been measured in the Generation R study.

Second, the specificity of the population in the present study should be considered when interpreting results. The women included in the Generation R study were healthier than the general population [44]. Based on the non-response analysis, we found that women excluded from the analysis were relatively younger, more often had an immigrant background, more often had a lower educational level, were more often single, and had more health conditions than women who were included in the analyses. Infants from women who were excluded from analyses more often had poorer birth outcomes, such as preterm birth and low birth weight. Given the selective non-response, it may be that we have underestimated the strength of the associations of the potential determinants of HRQoL. We have measured all the variables with validated scales and examinations. However, misclassification might have occurred, for example, by social desirability bias. Therefore, results should be interpreted with caution.

Third, the postnatal questionnaire was completed by women around two months after childbirth. Some items refer to a recall period of one month, while others, such as items about headache and loss of energy, refer to the first two weeks after childbirth; and hospital admission of the baby refers to the first week after birth. This should be taken into consideration.

## 5. Conclusions

We found that multiple factors are associated with worse physical and mental HRQoL of mothers two months after delivery. In particular, the presence of maternal psychopathology is associated with relatively poor physical and mental HRQoL after childbirth. Some risk factors such as loss of energy, headache, and maternal psychopathological symptoms may be manageable by adequate health and psychosocial care. Health professionals and clinicians should target these issues when they develop health interventions or provide education to help women to cope with these conditions.

## Figures and Tables

**Table 1:** Characteristics of mothers and infants (*n* = 4312).

Characteristics	Values *
**Demographic characteristics**	
Maternal age at enrollment, in years; mean (standard deviation)	31 (4.7)
Time since childbirth, in months, median (interquartile range)	2.8 (2.3–3.5)
Gender of infants, number (%)	
Girl	2145 (50)
Boy	2167 (50)
Maternal ethnic background, number (%)	
Dutch	2692 (63)
Other western	385 (9)
Non-western	1170 (28)
Maternal educational level, number (%)	
High	1307 (32)
Mid-high	1023 (25)
Mid-low	1190 (29)
Low	622 (15)
Marital status, number (%)	
Married/living together	3703 (90)
No partner	401 (10)
Household income, number (%)	
≤2200 euro/month	1280 (34)
>2200 euro/month	2474 (66)
Pregnancy-related characteristics	
Parity, *n* (%)	
Nullipara	2508 (59)
Multipara	1746 (41)
Twin birth, *n* (%)	53 (1)
Unplanned pregnancy, *n* (%)	844 (22)
Gestational weight gain, *n* (%)	
Inadequate weight gain	578 (20)
Adequate weight gain^1^	1024 (3)
Excessive weight gain	1307 (45)
Preeclampsia, *n* (%)	75 (2)
Pregnancy induced hypertension, *n* (%)	165 (4)
Gestational diabetes, *n* (%)	26 (1)
Hospitalization during pregnancy, *n* (%)	71 (2)
Delivery characterisitcs	
Mode of delivery, *n* (%)	
Spontaneous vaginal delivery	2987 (77)
Induced vaginal delivery	437 (11)
Elective cesarean delivery	199 (5)
Emergency cesarean delivery	276 (7)
Place of delivery, *n* (%)	
At home	771 (18)
At hospital	3433 (80)
In childbirth clinic or other places	95 (2)
Maternal health-related factors after childbirth	
Loss of energy (yes), *n* (%)	2151 (51)
Headache (yes), *n* (%)	590 (14)
Maternal psychopathology	
Lowest tertile	1517 (36)
Middle tertile	1322 (31)
Highest tertile	1414 (33)
Infant health-related factors	
Mecomium-stained amnoitic fluid, *n* (%)	624 (15)
Apgar score of <7 at 5 min, *n* (%)	43 (1)
Preterm birth, *n* (%)	219 (5)
Low birth weight^3^, *n* (%)	183 (4)
Small size for gestational age, *n* (%)	360 (8)
Intrauterine growth restriction, *n* (%)	60 (1)
Hospital admission of the baby in the first week, *n* (%)	706 (17)
HRQoL Summary scores	
Physical Component Summary score, mean (standard deviation)	44.8 (7)
Mental Component Summary score, mean (standard deviation)	54.2 (10)

* Values are means, standard deviations, median, interquartile range, numbers, and percentages for the whole study population.

**Table 2:** Multivariable associations with physical component summary score in the imputed dataset (*n* = 4312).

	Physical Component Summary Score	
	B (95% CI)	*p* Value
Maternal age at intake (in years)	**−0.10 (−0.15, −0.04)**	**<0.001**
Time since childbirth (in months)	**0.58 (0.34, 0.82)**	**<0.001**
Maternal Educational level		
High education	Reference	
Mid-high education	−0.47 (−1.06, 0.12)	0.12
Mid-low education	−0.46 (−1.09, 0.16)	0.15
Low education	0.13 (−0.66, 0.92)	0.74
Maternal ethnic background		
Dutch	Reference	
Other western	0.73 (−0.04, 1.49)	0.06
Non-western	−0.33 (−0.87, 0.22)	0.24
Household income		
High household income	Reference	
Low household income	**−0.68 (−1.32, −0.04)**	**0.04**
Marital status		
Married or living together	Reference	
Single	−0.33 (−1.16, 0.50)	0.44
Parity		
Nullipara	Reference	
Multipara	0.28 (−0.20, 0.76)	0.25
Pregnancy-induced hypertension		
No	Reference	
Yes	−0.26 (−1.01, 0.50)	0.49
Mode of delivery		
Spontaneous vaginal delivery	Reference	
Induced vaginal delivery	−0.58 (−1.32, 0.17)	0.13
Elective cesarean delivery	**−2.76 (−5.09, −0.42)**	**0.03**
Emergency cesarean delivery	**−1.91 (−2.97, −0.85)**	**<0.001**
Loss of energy		
No	Reference	
Yes	**−1.46 (−1.90, −1.02)**	**<0.001**
Headache		
No	Reference	
Yes	−0.58 (−1.22, −0.07)	0.08
Psychopathologic symptoms^1^		
Lowest tertile	**Reference**	
Middle tertile	**−1.53 (−2.05, −1.00)**	**<0.001**
Highest tertile	**−1.87 (−2.41, −1.33)**	**<0.001**
Hospital admission of the baby		
No	Reference	
Yes	**−0.67 (−1.26, −0.08)**	**0.03**

Table 2 is based on the imputed dataset. Values represent betas with 95% CIs (confidence intervals) and *p* values derived from multiple linear regression analyses. Bold print indicates the statistical significance. The significance level is *p* < 0.05. ^1^ Maternal psychopathology was measured by the brief symptom inventory.

**Table 3:** Multivariable associations with mental component summary score in the imputed dataset (*n* = 4312).

	Mental Component Summary Score	
	B (95% CI)	*p* Value
Maternal age at intake (in years)	**−0.14 (−0.20, −0.08)**	**<0.001**
Time since childbirth (in months)	−0.21 (−0.52, 0.10)	0.19
Infant’s gender		
Boy	Reference	
Girl	0.36 (−0.14, 0.87)	0.16
Maternal Educational level		
High education	Reference	
Mid-high education	−0.25 (−0.95, 0.44)	0.48
Mid-low education	−0.32 (−1.05, 0.41)	0.39
Low education	−0.78 (−1.74, 0.21)	0.12
Maternal ethnic background		
Dutch	Reference	
Other western	−0.45 (−1.35, 0.46)	0.33
Non-western	**−1.19 (−1.84, −0.54)**	**<0.001**
Household income		
High household income	Reference	
Low household income	**−1.28 (−2.00, −0.56)**	**0.001**
Marital status		
Married or living together	Reference	
Single	0.88 (0.−0.14, 1.62)	0.09
Unplanned pregnancy		
No	Reference	
Yes	**0.87 (0.11, 1.62)**	**0.02**
Location of delivery		
At home	Reference	
At hospital	−0.36 (−1.03,0.32)	0.30
At childbirth clinic or other places	−0.56 (−2.38, 1.26)	0.55
Loss of energy		
No	Reference	
Yes	**−1.60 (−2.15, −1.06)**	**<0.001**
Headache		
No	Reference	
Yes	**−1.51 (−2.26, −0.76)**	**<0.001**
Psychopathologic symptoms^1^		
Lowest tertile	Reference	
Middle tertile	**−2.98 (−3.60, −2.34)**	**<0.001**
Highest tertile	**−12.42 (−13.07, −11.78)**	**<0.001**
Preterm birth		
No	Reference	
Yes	−0.57 (−1.78, 0.64)	0.36
Hospital admission of the baby		
No	Reference	
Yes	−0.02 (−0.74, 0.70)	0.96

Table 3 is based on the imputed dataset. Values represent betas with 95% CIs (confidence intervals) and *p* values derived from multiple linear regression analyses. Bold print indicates the statistical significance. The significance level is *p* < 0.05. ^1^ Maternal psychopathology was measured by the brief symptom inventory.

## Supplementary Materials

The following are available online at https://www.mdpi.com/1660-4601/16/18/3231/s1, Figure S1. Flow chart of the study population, Table S1. Characteristics of the study population (*n* = 4312), Table S2. Differences in Physical and Mental Component Summary scores across subgroups (*n* = 4312), Supplementary Table S3. Multivariable associations with Physical and Mental Component Summary scores in the non-imputed datasets, Supplementary Table S4. Characteristics of the study population (*n* = 4321) and the population excluded from analyses (*n* = 5466)
