# Supplementary material for: Determinants of Maternal Health-Related Quality of Life after Childbirth: The Generation R Study
Source: Int J Environ Res Public Health. 2019 Sep 4;16(18):3231. doi: 10.3390/ijerph16183231 (PMC6765914; doi:10.3390/ijerph16183231)
Supplement: Supplementary file 1 [file ijerph-16-03231-s001.pdf]

Supplementary Table S1. Differences in Physical and Mental Component Summary scores across subgroups (n=4312)

	Physical Component Summary score			Mental Component Summary score		
	Mean (SD)	P value	Effect size	Mean (SD)	P value	Effect size
Infant gender		0.95	0.01		0.02	0.07
Girl (n=2145)	44.8 (7.2)			54.6 (9.9)		
Boy (n=2167)	44.7 (7.4)			53.9 (10.6)		
Maternal educational level		0.02	0.11		<0.001	0.24
High (n=1307)	45.1 (7.0)			55.7 (8.5)		
Mid-high (n=1023)	44.4 (7.4)			54.4 (10.0)		
Mid-low (n=1190)	44.4 (7.5)			53.7 (10.9)		
Low (n=622)	45.2 (7.0)			52.9 (11.4)		
Maternal ethnic background		0.03	0.14		<0.001	0.31
Dutch (n=2692)	44.8 (7.0)			55.4 (9.1)		
Other western (n=385)	45.5 (7.1)			54.0 (10.2)		
Non-western (n=1170)	44.4 (7.8)			51.7 (12.0)		
Marital status		0.04	0.10		<0.001	0.21
Married/living together (n=3703)	44.8 (7.2)			54.5 (10.0)		
No partner (n=401)	44.0 (8.0)			52.0 (11.7)		
Household income		0.04	0.07		<0.001	0.33
≤2200 euro/month (n=1280)	44.4 (7.5)			51.8 (11.7)		
>2200 euro/month (n=2474)	44.9 (7.1)			55.7 (8.9)		
Parity		0.04	0.07		0.45	0.02
Nullipara (n=2508)	44.5 (7.4)			54.3 (10.2)		
Multipara (n=1746)	45.0 (7.2)			54.1 (10.3)		
Twin birth		0.29	0.15		0.11	0.20
Yes (n=53)	45.8 (6.8)			51.9 (11.6)		
No (n=4259)	44.7 (7.3)			54.2 (10.2)		
Unplanned pregnancy		0.93	0.01		<0.001	0.25
Yes (n=844)	44.7 (7.5)			52.1 (11.5)		
No (n=3054)	44.8 (7.2)			55.0 (9.6)		
Gestational weight gain		0.49	0.06		0.24	0.08
Adequate weight gain[1] (n=1024)	44.8 (7.3)			54.8 (9.7)		
Inadequate weight gain (n=578)	45.1 (7.0)			54.0 (10.5)		
Excessive weight gain (n=1307)	44.6 (7.3)			54.2 (10.4)		
Preeclampsia		0.42	0.07		0.22	0.13
Yes (n=75)	44.2 (8.5)			52.8 (10.9)		
No (n=3764)	44.8 (7.2)			54.2 (10.2)		
Pregnancy-induced hypertension		0.004	0.21		0.64	0.04
Yes (n=165)	43.2 (7.7)			54.6 (11.0)		
No (n=3764)	44.8 (7.2)			54.2 (10.2)		
Gestational diabetes		0.40	0.15		0.85	0.03
Yes (n=26)	43.5 (7.9)			54.6 (12.2)		
No (n=4170)	44.7 (7.3)			54.2 (10.3)		
Hospitalization during pregnancy		0.10	0.17		0.05	0.18
Yes (n=71)	43.4 (8.1)			52.1 (12.5)		

No (n=3674)	44.8 (7.2)			54.5 (10.0)		
Mode of delivery		<0.001	0.49		0.26	0.12
Spontaneous vaginal delivery (n=2987)	45.2 (7.0)			54.4 (9.9)		
Induced vaginal delivery (n=437)	44.2 (7.6)			53.6 (11.2)		
Elective cesarean delivery (n=199)	41.1 (8.4)			54.1 (10.9)		
Emergency cesarean delivery (n=276)	42.3 (8.2)			53.2 (10.2)		
Location of delivery		0.01	0.10		<0.001	0.33
At home (n=771)	45.4 (6.8)			55.5 (8.2)		
At hospital (n=3433)	44.6 (7.3)			54.0 (10.6)		
In childbirth clinics or other places (n=95)	44.6 (8.3)			51.7 (11.6)		
Loss of energy		<0.001	0.26		<0.001	0.24
Yes (n=2152)	43.8 (7.7)			52.8 (10.9)		
No (n=2043)	45.8 (6.6)			56.0 (8.9)		
Headache		<0.001	0.14		<0.001	0.30
Yes (n=590)	43.8 (7.7)			51.2 (12.2)		
No (n=3629)	44.9 (7.2)			54.8 (9.7)		
Maternal psychopathology[2]		<0.001	0.28		<0.001	1.10
Lowest tertile	46.1 (5.8)			59.8 (4.7)		
Middle tertile	44.3 (7.2)			56.4 (6.2)		
Highest tertile	43.7 (8.5)			46.3 (12.3)		
Mecomium-stained amnoitic fluid		0.07	0.08		0.64	0.02
Yes (n=624)	45.2 (6.7)			54.0 (10.6)		
No (n=3519)	44.6 (7.4)			54.2 (10.2)		
Apgar score of <7 at 5 minutes, n (%)		0.35	0.14		0.19	0.16
Yes (n=43)	43.7 (7.4)			52.2 (12.0)		
No (n=4092)	44.7 (7.3)			54.2 (10.2)		
Preterm birth		0.18	0.10		0.003	0.18
Yes (n=219)	45.4 (7.3)			52.2 (11.7)		
No (n=4092)	44.7 (7.3)			54.3 (10.2)		
Low birth weight[3]		0.54	0.19		0.47	0.08
Yes (n=183)	45.4 (7.3)			53.6 (10.4)		
No (n=4129)	44.7 (7.3)			54.2 (10.2)		
Small size for gestational age		0.14	0.08		0.20	0.07
Yes (n=360)	45.3 (6.9)			53.5 (10.6)		
No (n=3948)	44.7 (7.3)			54.2 (10.2)		
Intrauterine growth restriction (IUGR)		0.21	0.16		0.48	0.09
Yes (n=60)	45.9 (7.2)			53.2 (11.0)		
No (n=4170)	44.7 (7.3)			54.2 (10.3)		
Hospital admission of the baby		<0.001	0.17		0.002	0.12
Yes (n=706)	43.7 (7.8)			53.1 (10.7)		
No (n=3511)	45.0 (7.2)			54.4 (10.1)		

[1]Adequate weight gain was defined depending on pre-pregnancy BMI categories. In accordance with the Institute of Medicine guideline, underweight women (BMI <18.5 kg/m2) should gain 12.5-18 kg during pregnancy, normal weight women (BMI 18.5–24.9 kg/m2) should gain 11.5-16 kg, overweight women (BMI 25.0–29.9 kg/m2) should gain 7-11.5 kg, and obese women (BMI ≥ 30 kg/m2) should gain 5-9 kg.

[2] Maternal psychopathology was measured by the Brief Symptom Inventory.
[3]Low birth weight was defined as the birth weight is lower than 2500 grams.
Effect size (d) calculated by dividing the difference in mean scores between subgroups by the largest standard deviation and interpreted as: $0.2 \leq d < 0.5$ small difference, $0.5 \leq d <0.8$ moderate difference, and $d \geq 0.8$ large difference.

Supplementary Table S2. Multivariable associations with Physical and Mental Component Summary scores in the non-imputed datasets

	Physical Component Summary score (n=2727)		Mental Component Summary score (n=2859)	
	B (95% CI)	P value	B (95% CI)	P value
Maternal age at intake (in years)	**-0.10 (-0.17, -0.04)**	**0.003**	**-0.12 (-0.19, -0.04)**	**0.002**
Time after delivery (in months)	**0.66 (0.39, 0.94)**	**<0.001**	-0.11 (-0.42, 0.20)	0.49
Infant's gender				
Boy			Reference	
Girl			0.37 (-0.22, 0.96)	0.22
Maternal Educational level				
High education	Reference		Reference	
Mid-high education	**-0.88 (-1.57, -0.18)**	**0.01**	-0.02 (-0.76, 0.80)	0.96
Mid-low education	-0.68 (-1.42, 0.06)	0.07	-0.20 (-1.02, 0.62)	0.64
Low education	0.15 (-0.85, 1.14)	0.77	-0.91 (-2.02, 0.20)	0.11
Maternal ethnic background				
Dutch	Reference		Reference	
Other western	0.39 (-0.60, 1.38)	0.44	-0.38 (-1.48, 0.72)	0.50
Non-western	-0.26 (-0.94, 0.42)	0.46	-0.74 (-1.51, 0.02)	0.06
Household income				
High household income	Reference		Reference	
Low household income	-0.37 (-1.07, 0.32)	0.29	**-0.99 (-1.77, -0.21)**	**0.01**
Marital status				
Married or living together	Reference		Reference	
Single	-0.24 (-1.26, 0.78)	0.65	0.31 (-0.88, 1.49)	0.61
Parity				
Nullipara	Reference			
Multipara	0.14 (-0.45, 0.73）	0.64		
Unplanned pregnancy				
No			Reference	
Yes			0.61 (-0.19, 1.40)	0.13
Pregnancy-induced hypertension				
No	Reference			
Yes	-0.95 (-2.23, 0.33)	0.15		
Mode of delivery				
Spontaneous vaginal delivery	Reference			
Induced vaginal delivery	**-1.09 (-2.01, -0.17)**	**0.02**		
Elective cesarean delivery	**-3.75 (-5.06, -2.45)**	**<0.001**		
Emergency cesarean delivery	**-2.40 (-3.48, -1.33)**	**<0.001**		
Location of delivery				
At home	Reference		Reference	
At hospital	0.04 (-0.69, 0.78)	0.91	0.20 (-0.95,0.56)	0.62
At childbirth clinic or other places	-1.52 (-3.56, 0.52)	0.14	-1.12 (-3.24, 1.01)	0.30
Loss of energy				
No	Reference		Reference	
Yes	**-1.51 (-2.06, -0.97)**	**<0.001**	**-1.54 (-2.15, -0.93)**	**<0.001**
Headache				
No	Reference		Reference	
Yes	-0.73 (-1.50, 0.04)	0.06	**-1.47 (-2.33, -0.61)**	**0.001**
Maternal psychopathology				
Lowest tertile	reference		reference	
Middle tertile	**-1.40 (-2.04, -0.76)**	**<0.001**	**-3.00 (-3.72, -2.28)**	**<0.001**

Highest tertile	**-1.73 (-2.40, -1.06)**	**<0.001**	**-11.55 (-12.30, -10.79)**	**<0.001**
Preterm birth				
No			Reference	
Yes			-0.33 (-1.86, 1.21)	0.68
Hospital admission of the baby				
No	Reference		Reference	
Yes	**-1.04 (-1.78, -0.30)**	**0.006**	-0.11 (-0.97, 0.75)	0.80

The above table is based on the non-imputed data. Values represent betas with 95% CIs (confidence intervals) and p values derived from multiple linear regression analyses.

The significance level is $p<0.05$

[1] Maternal psychopathology was measured by the Brief Symptom Inventory.

Supplementary Table S3. Characteristics of the study population (n=4321) and the population excluded from analyses (n=5466)

Characteristics	Study population (n=4312)	Exclude population (n=5466)	P value
**Mother/ infant demographic characteristics**			
Maternal age at enrollment, in years; mean (standard deviation)	31.0 (4.7)	29.0 (5.7)	<0.001
missing	0	3	
Time after delivery, in months, median (interquartile range)	2.8 (2.3 – 3.5)	2.9 (2.3 – 3.8)	0.004
Range	0.4 – 6.0	0.8 – 6.0	
missing	*350*	*4811*	
Gender of infants, n (%)			0.49
Girl	2145 (49.7)	2604 (49.0)	
Boy	2167 (50.3)	2708 (51.0)	
missing	*0*	*154*	
Maternal ethnic background, n (%)			<0.001
Dutch	2692 (63.4)	1853 (38.3)	
Other western	385 (9.1)	391 (8.1)	
Non-western	1170 (27.5)	2592 (53.6)	
missing	*65*	*630*	
Maternal educational level, n (%)			<0.001
High	1307 (31.6)	698 (15.8)	
Mid-high	1023 (24.7)	632 (14.3)	
Mid-low	1190 (28.7)	1436 (32.5)	
Low	622 (15.0)	1648 (37.3)	
missing	*170*	*1052*	
Marital status, n (%)			<0.001
Married/living together	3703 (90.2)	3623 (81.2)	
No partner	401 (9.8)	837 (18.8)	
missing	*208*	*1006*	
Household income, n (%)			<0.001
≤2200 euro/month	1280 (34.1)	1784 (60.3)	
>2200 euro/month	2474 (65.9)	1173 (39.7)	
missing	*558*	*2400*	
**Pregnancy-related characteristics**			
Parity, n (%)			<0.001
Nullipara	2508 (59.0)	2669 (51.9)	
Multipara	1746 (41.0)	2476 (48.1)	
missing	*58*	*510*	
Twin birth, n (%)	53 (1.2)	70 (1.3)	0.86
missing			
Unplanned pregnancy, n (%)	844 (21.7)		
missing	*414*		
Gestational weight gain, n (%)			0.10
Inadequate weight gain	578 (19.9)	104 (23.9)	
Adequate weight gain[1]	1024 (35.2)	154 (35.4)	
Excessive weight gain	1307 (44.9)	177 (40.7)	
missing	*1403*	*5031*	
Preeclampsia, n (%)	75 (2.0)	112 (2.6)	0.06
missing	*473*	*1136*	

Pregnancy induced hypertension, n (%)	165 (4.2)	153 (3.5)	0.11
missing	*370*	*1095*	
Gestational diabetes, n (%)	26 (0.6)	77 (1.5)	<0.001
missing	*116*	*483*	
Hospitalization during pregnancy, n (%)	71 (1.9)	90 (3.0)	0.003
missing	*567*	*2492*	
**Delivery characteristics**			
Mode of delivery, n (%)			0.03
Spontaneous vaginal delivery	2987 (76.6)	3360 (74.1)	
Induced vaginal delivery	437 (11.2)	575 (12.7)	
Elective cesarean delivery	199 (5.1)	227 (5.0)	
Emergency cesarean delivery	276 (7.1)	374 (8.2)	
missing	*413*	*930*	
Location of delivery, n (%)			<0.001
At home	771 (17.9)	452 (8.7)	
At hospital	3433 (79.9)	4552 (87.3)	
In childbirth clinic or other places	95 (2.2)	211 (4.0)	
missing	*13*	*251*	
**Maternal postpartum health-related factors**			
Loss of energy (yes), n (%)	2151 (51.3)	285 (42.2)	<0.001
Missing	*118*	*4791*	
Headache (yes), n (%)	590 (14.0)	116 (17.1)	0.04
missing	*93*	*4786*	
Maternal psychopathology			0.001
Lowest tertile	1517 (35.7)	200 (30.8)	
Middle tertile	1322 (31.1)	186 (28.6)	
Highest tertile	1414 (33.2)	264 (40.6)	
missing	*59*	*4816*	
**Infant health-related factors**			
Mecomium-stained amnoitic fluid, n (%)	624 (15.1)	733 (15.0)	0.95
missing	*169*	*582*	
Apgar score of <7 at 5 minutes, n (%)	43 (1.0)	57 (1.2)	0.55
missing	*177*	*645*	
Preterm birth, n (%)	219 (5.1)	404 (7.7)	<0.001
missing	*1*	*239*	
Low birth weight[3], n (%)	183 (4.2)	335 (6.4)	<0.001
Small size for gestational age, n (%)	360 (8.4)	232	
missing	*4*		
Intrauterine growth restriction (IUGR), n (%)	60 (1.4)	99 (2.0)	0.04
missing	*129*	*506*	
Hospital admission of the baby, n (%)	706 (16.7)	153 (17.6)	0.55
missing	*95*	*4597*	

[1]Adequate weight gain was defined depending on pre-pregnancy BMI categories. In accordance with the Institute of Medicine guideline, underweight women (BMI <18.5 kg/m2) should gain 12.5-18 kg during pregnancy, normal weight women (BMI 18.5–24.9 kg/m2) should gain 11.5-16 kg,

overweight women (BMI 25.0–29.9 kg/m2) should gain 7-11.5 kg, and obese women (BMI ≥ 30 kg/m2) should gain 5-9 kg.

[2] Maternal psychopathology was measured by the Brief Symptom Inventory.

[3]Low birth weight was defined as the birth weight is lower than 2500 grams.
